# Contraceptive-induced impairment: a rodent model study of levonorgestrel and DMPA

**DOI:** 10.5935/1518-0557.20250041

**Published:** 2025

**Authors:** F.A. Akinpelu, O.L. Apanisile, B.M. Oguntimilehin, E.N. Akang

**Affiliations:** 1 Department of Anatomy, Faculty of Basic Medical Sciences, College of Medicine, University of Lagos, Lagos, Nigeria; 2 Department of Anatomy, Achiever’s College of Nursing Sciences, Akure, Ondo State, Nigeria; 3 Department of Anatomy, Faculty Basic Medical Sciences, University of Ilorin, Ilorin, Nigeria; 4 Department of Anatomy, Faculty of Basic Medical Sciences, Redeemer’s University, Ede, Osun State, Nigeria; 5 Department of Cell and Developmental Biology, Center of Anatomy and Cell Biology, Medical University of Vienna, 1090 Vienna, Austria

**Keywords:** contraceptives, levonorgestrel, depot-medroxyprogesterone acetate, oestrous cycle, fecundity, infertility

## Abstract

**Objective:**

Hormonal contraceptives are widely used to alter the hypothalamic-pituitary-gonadal (HPG) axis, inhibiting ovulation, and altering reproductive hormone levels. Understanding their effects on estrous cycle regulation and fecundity in animal models is essential for evaluating their contraceptive mechanisms and long-term reproductive impact. This study aims to investigate the effects of Levonorgestrel and Depot-Medroxyprogesterone Acetate (DMPA) on estrous cycle phases, hormonal disruptions, and fecundity in rodent models, providing insights into their contraceptive efficacy and potential reproductive consequences.

**Methods:**

Fifteen adults female Wistar rats were divided into three groups: Control (water only), Levonorgestrel-treated (0.7mL oral administration), and DMPA-treated (0.02mL intramuscular injection). Vaginal cytology was used to track estrous cycle phases before, during, and after treatment. Mating success and fecundity were assessed by pairing treated females with males and recording pregnancy rates and litter sizes.

**Results:**

Both Levonorgestrel and DMPA significantly disrupted estrous cycle regularity. The estrus phase duration was notably shortened in treated groups, while the diestrus phase was prolonged, especially in the DMPA group (*p*<0.0001). Mating success was significantly reduced, with only 40% of DMPA-treated and 60% of Levonorgestrel-treated females mating successfully, compared to 100% in the control group. Litter numbers were also significantly lower in treated groups compared to controls.

**Conclusions:**

These findings suggest that prolonged use of Levonorgestrel or DMPA may significantly alter the oestrous cycle, potentially impairing fertility and delaying reproductive recovery. Further studies are needed to explore the long-term consequences of contraceptive-induced cycle disruptions.

## INTRODUCTION

Contraceptives have significantly transformed reproductive health, empowering individuals to manage fertility and family planning effectively. Among the widely used methods are hormonal contraceptives such as Depot-Medroxyprogesterone Acetate (DMPA) and Levonorgestrel. DMPA, a long-acting injectable progestin, is administered intramuscularly every three months to prevent ovulation and create an inhospitable uterine environment for implantation (World Health Organization, 2016). Levonorgestrel, commonly used in emergency contraceptive pills, also known as a morning-after pill, acts primarily by inhibiting ovulation and altering cervical mucus to prevent sperm penetration (Gemzell-Danielsson, 2010; Glasier *et al.*, 2010). These contraceptives are highly effective and convenient; however, their potential effects on fertility recovery and reproductive health remain areas of concern.

Reproductive physiology relies on intricate hormonal regulation, and any disruption may affect the Estrous cycle in animals or the menstrual cycle in humans. The Estrous cycle in female rodents is analogous to the human menstrual cycle in its hormonal underpinnings; it serves as a reliable model for studying reproductive health (Marcondes *et al.*, 2002; Ajayi & Akhigbe, 2020; Zavala *et al.*, 2020). Contraceptives like DMPA and Levonorgestrel may interfere with these hormonal pathways, potentially altering the regularity of the Estrous cycle and reducing fecundity (Teal & Edelman, 2021). Such alterations can manifest as prolonged cycle phases, disrupted ovulation, or reduced litter size, raising questions about their long-term safety for women intending to conceive after contraceptive use (Lohmiller & Swing, 2006; Girum & Wasie, 2018).

Despite extensive research on contraceptive efficacy, studies investigating their impacts on fertility recovery are sparse. This gap shows the need for experimental studies to elucidate the reproductive effects of contraceptives, particularly progestin-based ones like DMPA and Levonorgestrel; understanding these effects is important in improving contraceptive safety profiles and provide users with accurate information about fertility restoration.

This study aims to investigate the effects of DMPA and Levonorgestrel on the estrous cycle and fecundity in female Wistar rats. By monitoring the estrous cycle phases and evaluating fecundity outcomes such as mating success and litter size, this study seeks to contribute valuable insights into the reproductive implications of these contraceptives.

## MATERIALS AND METHODS

### Experimental Animals

Fifteen adult female Wistar rats (Rattus norvegicus) obtained from the laboratory animal house at the College of Health Sciences, Bowen University, Iwo, Nigeria were randomly selected. The rats weighed 130 g- 150 g and were about 10 - 12 weeks old. The rats were housed in standard plastic cages with sawdust bedding under controlled conditions of temperature (22±2°C), humidity (50-70%), and a 12-hour light/dark cycle. They were acclimatized for two weeks prior to the experiment. They were provided with unrestricted access to rat chow and clean water.

### Experimental Design

The rats were randomly divided into three groups (n = 5 per group) as follows:

- **Group 1 (Control):** Received fresh water ad libitum without any contraceptive treatment.
- **Group 2 (DMPA-treated):** Received 0.02 mL of DMPA (Depot-Provera) via intramuscular injection.
- **Group 3 (Levonorgestrel-treated):** Received 0.7 mL of Levonorgestrel solution (prepared from Postinor-2 tablets) via oral administration using an oral cannula.

The groups were subjected to the same feeding regime and also weighed weekly. The treatment period lasted four weeks, during which the estrous cycle phases and fecundity parameters were monitored.

### Preparation and Administration of Drugs

- **Depot-Medroxyprogesterone Acetate (DMPA):** A commercially available DMPA injectable contraceptive was used. A dose of 0.02 mL was drawn using an insulin syringe and administered intramuscularly into the thigh muscles of the rats.
- **Levonorgestrel:** Postinor-2 tablets containing 1.5 mg of levonorgestrel per tablet were dissolved in 100 mL of distilled water to create a solution. The solution was stored in an airtight container and administered orally at a dose of 0.7 mL per rat using oral gavage.

### Monitoring of Estrous Cycle

The estrous cycle was monitored daily throughout the treatment period using vaginal cytology. Vaginal secretions were collected between 6:30 a.m. and 7:30 a.m. by gently inserting a plastic pipette filled with 10 μL of normal saline (0.9% NaCl) into the vaginal canal without causing discomfort. The fluid was transferred onto a clean glass slide, smeared, and air-dried. Unstained smears were examined under a light microscope at 10× and 40× magnifications to identify the phases of the estrous cycle (proestrus, estrus, metestrus, and diestrus) based on the proportion of epithelial cells, cornified cells, and leukocytes (Marcondes *et al.*, 2002).

### Estrous Cycle Phases

- **Proestrus:** Predominance of nucleated epithelial cells.
- **Estrus:** Predominance of cornified epithelial cells.
- **Metestrus:** Mixed population of leukocytes, cornified, and nucleated epithelial cells.
- **Diestrus:** Predominance of leukocytes.
- The length of each cycle phase and the overall cycle duration were recorded for all groups.

### Fecundity Assessment

After the treatment period, all female rats were paired with untreated fertile male rats (1:1 ratio) for seven days. Mating success was confirmed by the presence of a vaginal plug, indicative of copulation. Pregnant females were separated and allowed to deliver, and the following parameters were recorded:

- Number of pregnancies per group (mating success rate).
- Average litter size per group.

### Ethical Considerations

The use of the animals was in accordance with the national law on animal care and use (Zimmermann, 1983) and approved by the Experimental Ethics Committee on Animals Use of Bowen University, Iwo, Nigeria. All efforts were made to minimize animal suffering, and humane endpoints were applied when necessary.

### Statistical Analysis

Statistics GraphPad prism software program version 5.0 (GraphPad Software, San Diego, California, USA) was used for statistical analyses. One-way ANOVA with Turkey’s multiple comparison *post-hoc* test was used.

## RESULTS

**Figure 1:**
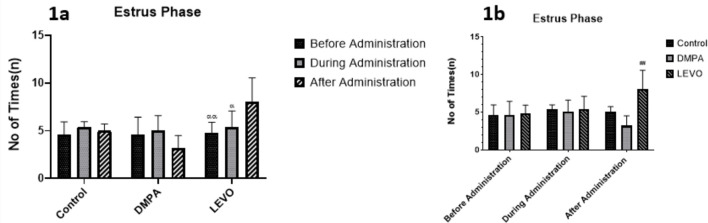
A. Comparing the differences in the duration of Estrus phase before, during, and after administration within each group. α denotes significant difference, *p*<0.05 compared with “After Administration”, αα denotes significant difference *p*<0.01 compared with “After administration”. B. Comparing the differences in the duration of Estrus phase between groups before, during, and after administration. ## denotes significant difference *p*<0.01 compared with “Control group”.

In this study, Figure 1a shows substantial changes in estrus phase duration across all treated groups, with levonorgestrel-treated rats demonstrating a significant difference before and after treatment (*p*=0.0054), and also showing a significant difference during and after treatment (*p*=0.0266). As shown in Fig. 1b, the levonorgestreltreated groups also exhibited a significant difference when compared with the control group (*p*<0.01).

**Figure 2:**
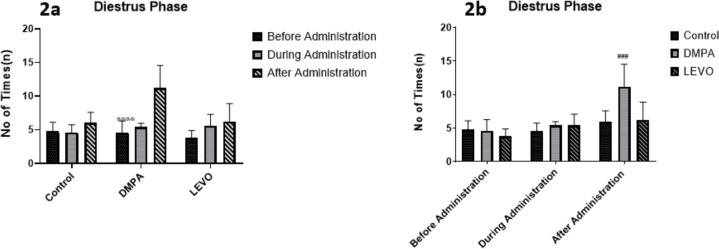
A. Comparing the differences in the duration of Diestrus phase before, during, and after administration within each group. αααα denotes significant difference *p*<0.0001 compared with After administration. B. Comparing the differences in the duration of Diestrus phase between groups before, during, and after administration. ### denotes significant difference *p*<0.001 compared with Control group.

Figure 2a, depicts a marked difference in the diestrus phase duration within the DMPA-treated group before and after administration, showing a highly significant difference (*p*<0.0001). Similarly, Figure 2b, highlights a significant difference between the DMPA-treated group and the control group (*p*<0.001).

**Figure 3:**
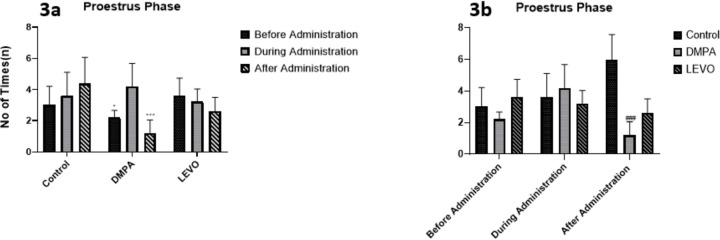
A. Comparing the differences in the duration of Proestrus phase before, during, and after administration within each group. * denotes significant difference *p*<0.05 compared with During administration, *** denotes significant difference *p*<0.001 compared with during administration. B. Comparing the differences in the duration of Proestrus phase between groups before, during, and after administration. ### denotes significant difference *p*<0.001 compared with control group.

Figure 3a, presents significant variations in the proestrus phase across administration periods, with a notable decrease during administration (*p*<0.05) and a highly significant reduction after administration (*p*<0.001). Comparisons between groups, as shown in Figure 3b, indicate a significant difference in proestrus phase duration in the treated groups relative to the control group (*p*<0.001).

**Figure 4:**
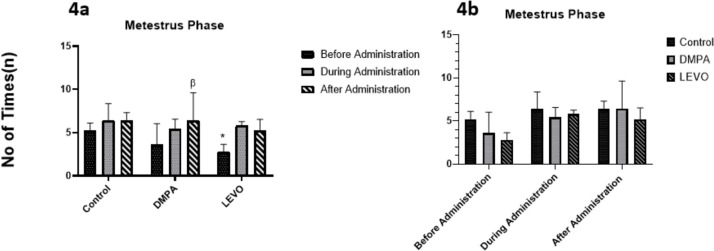
A. Comparing the differences in the duration of Metestrus phase before, during, and after administration within each group. * denotes significant difference *p*<0.05 compared with “during administration”, β denotes significant difference *p*<0.05 compared with before administration”. B. Comparing the differences in the duration of Metestrus phase between groups before, during, and after administration.

Figure 4a showed that the Metestrus phase varied significantly across the three groups. There was significant increase in the metestrus phase during administration and after administration in the DMPA- and Levonorgestrel- Treated groups when compared with before administration as shown in figure 4b.

**Figure 5:**
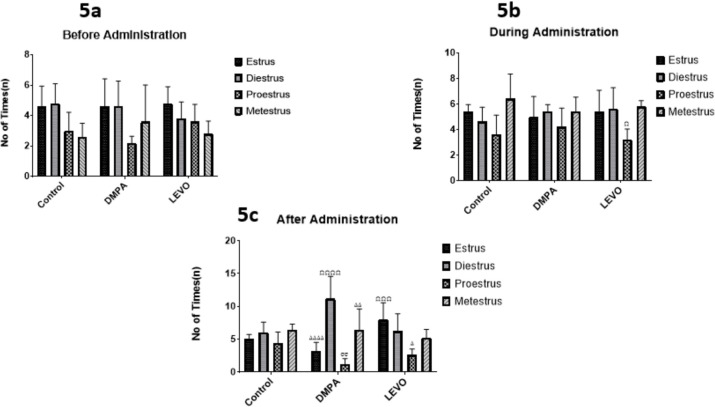
A. Comparing the differences in the duration of the Oestrus cycle across all groups before administration. B. Comparing the differences in the duration of the Oestrus cycle across all groups during administration. Ω denotes significant difference *p*<0.05 when compared with estrus, diestrus, and mestestrus phases. C. Comparing the differences in the duration of the Oestrus cycle across all groups after administration. ΩΩΩ denotes significant difference *p*<0.001 compared with Proestrus phase, ΩΩΩΩ denotes significant difference *p*<0.0001 compared with Proestrus phase, ∆ denotes significant difference *p*<0.05 compared with Diestrus Phase, ∆∆ denotes significant difference *p*<0.01 compared with Diestrus phase, ∆∆∆∆ denotes significant difference *p*<0.0001 compared with Diestrus phase, σσ denotes significant difference *p*<0.01 compared with Metestrus phase.

Figure 5a-c illustrate variations in the duration of the estrous cycle across all groups. Significant differences were observed during administration (*p*<0.05), with further highly significant differences after administration, particularly in the proestrus phase (*p*<0.001, *p*<0.0001) and diestrus phase (*p*<0.05 to *p*<0.0001). While the control group remains remained stable and consistent.

The result in Table 1 showed that the mating success rate and average litter size varied significantly across the three groups. Mating success rate declined significantly in DMPA- and Levornorgestrel- Treated groups (40% and 60%, respectively) compared to the control group (100%), with a corresponding reduction in average litter size (3±1 and 5±2 *vs*. 8±1 in control). These findings indicate that both hormonal treatments significantly impair reproductive outcomes compared to the control group.

**Table 1:** Mating Success Rate across all groups: Values are mean±standard error of the mean, ^*^ *p*<0.05 *vs*. Control Group.

Group	Mating Success Rate (%)	Average Litter Size	Chi-Square Value	p-Value
Control	100	8±1	Reference Group	-
DMPA-Treated	40	3±1	*χ*^2^ = 6.89	*p*<0.05
Levonorgestrel-Treated	60	5±2	*χ*^2^ = 4.56	*p*<0.05

## DISCUSSION

### Effect on oestrous cycle

Levonorgestrel and Depot Medroxyprogesterone Acetate (DMPA) are progestin-based contraceptives that suppress the hypothalamic-pituitary axis by reducing gonadotropin-releasing hormone (GnRH) and luteinizing hormone (LH) release. This inhibits ovulation and lowers estrogen levels, which likely shortens or disrupts the estrus phase. In this study, the estrus phase, which is characterized by high estrogen levels and sexual receptivity, was altered, resulting in less estrogen production and changing its timing. Our findings on the impact of LEVO and DMPA on estrogen are in concordance with Sitruk-Ware (2004; 2008), who stated that progestins suppress the hypothalamic-pituitary-ovarian axis, lowering estrogen levels and disrupting the estrus phase.

The Diestrus phase, marked by high progesterone and low estrogen levels, was prolonged by DMPA, a long-acting progestin that maintains elevated progesterone levels. The observed diestrus prolongation reflects sustained progesterone dominance from DMPA’s suppression of follicular turnover. As demonstrated by Kaunitz & Rosenfield (1993), DMPA’s long-acting prostogenic activity maintains luteal-phase conditions through GnRH suppression by inhibiting the arcuate nucleus (Clarkson & Herbison, 2016), LH surge blockade (disrupting POA’s(preoptic area) estrogen-positive feedback (Moenter *et al.*, 2003); and direct endometrial progesterone receptor activation (Sitruk-Ware, 2004; 2008).

The administration of levonorgestrel and DMPA significantly disrupted the proestrus phase duration as shown in (Figure 3). The oestrous cycle is regulated by the hypothalamic-pituitary-ovaria (HPO)axis, where arcuate and preoptic hypothalamic nuclei secrete pulsatile GnRH to drive pituitary FSH/LH release, orchestrating follicular maturation and steroidogenesis (Clarkson & Herbison, 2016). During proestrus, rising estrogen from follicles stimulates the POA to amplify GnRH, triggering the preovulatory LH surge required for ovulation (Freeman, 2006). Post ovulation, progesterone from the corpus luteum suppresses gonadotropins, initiating metestrus and diestrus (Norman *et al.*, 2007). Levonorgestrel and DMPA disrupt this axis by binding progesterone receptors in the ARC/POA, inhibiting GnRH pulsatility and LH surges (Sitruk-Ware, 2004; 2008), thereby delaying follicular maturation (evidenced by prolonged proestrus: Levo *p*=0.0008; DMPA *p*=0.004) and sustaining progesterone dominance. This mimics diestrus-like hormonal stasis, suppressing phase transitions. However, species-specific compensatory mechanisms (eg., primate kisspeptin signalling) may mitigate such disruptions (Evans & Anderson, 2012), underscoring translational nuances in contraceptive research.

The Metestrus phase was altered by both contraceptives; DMPA and Levonorgestrel indicated a significant difference before, during, and after administration. Progestins sustain the Metestrus phase, which is characterized by rising progesterone and falling estrogen, by maintaining progesterone activity. According to Norman *et al.*, 2007, progesterone stabilizes endometrial receptivity, extending the luteal phase, which coincides with ovulation. This most certainly accounts for the DMPA group's elongation. However, some research contends that, particularly in animals with shorter cycles, menstruation is less sensitive to hormone manipulation (Freeman, 2006; Evans & Anderson, 2012).

In this study, DMPA had a prolonged duration of diestrus phase which subsequently might have lowered the frequency at which estrus phase occurred several cycles after administration. In this same DMPA group, estrus phase had a short duration. This could suggest that the administered contraceptives had an effect on reducing the frequency of ovulation which is estrus leading to impairment of fertility.

The prolonged diestrus phase observed in DMPA-treated and Levonorgestrel-treated rats suggests significant hormonal alterations, potentially attributable to the mechanism of action of progestin-based contraceptives. Both DMPA and Levonorgestrel suppress gonadotropin secretion, inhibiting follicular maturation and ovulation (Gemzell-Danielsson, 2010; Jacobstein & Polis, 2014; Teal & Edelman, 2021). The extended diestrus phase, a luteal phase equivalent, indicates persistent progesterone dominance, delaying the transition to subsequent follicular development. These findings align with previous studies highlighting the inhibitory effects of progestins on ovarian function in both human and animal models (Marcondes *et al.*, 2002).

The alterations observed in the oestrus cycle is also buttressed by our findings on fecundity. In this study, the administration of Depot Medroxyprogesterone Acetate (DMPA) and Levonorgestrel significantly reduces mating success and fecundity compared to the control group. The decline in mating success was more pronounced in the DMPA-treated group, where only 40% of the subjects successfully mated, compared to 60% in the Levonorgestrel-treated group and 100% in the control group. This suggests that DMPA exerts a stronger inhibitory effect on reproductive behaviour than Levonorgestrel.

The reduction in mating success may be attributed to hormonal alterations that interfere with sexual behavior and reproductive physiology. DMPA, a long-acting progestin, is known to suppress gonadotropin secretion by acting on the hypothalamic-pituitary-gonadal (HPG) axis, leading to a decrease in luteinizing hormone (LH) and follicle-stimulating hormone (FSH) levels (Schwallie & Assenzo, 1973; 1974). This suppression prevents ovulation and disrupts the normal estrous cycle, ultimately reducing sexual receptivity. Additionally, studies in non-human primates have demonstrated that DMPA administration leads to long-term suppression of sexual behavior and pheromone signaling, further diminishing mating success (Goldzieher & Kraemer, 1972; Muller, 2017).

Several mechanisms may account for the observed reduction in fecundity. One primary factor is the suppression of follicular development due to decreased gonadotropin secretion. Both DMPA and Levonorgestrel act on progesterone receptors to suppress ovulation by reducing LH and FSH levels, leading to follicular atresia and a lower number of ovulated eggs (Speroff & Darney, 2011). Additionally, both contraceptives induce endometrial changes that make the uterine lining less receptive to implantation. DMPA, in particular, causes significant thinning of the endometrium, thereby preventing successful embryo attachment (Erkkola & Landgren, 2005). While Levonorgestrel also affects the endometrium, its impact is less pronounced, which may explain the comparatively higher litter size observed in this group.

Another important factor that may have contributed to reduced fecundity is the alteration of cervical mucus composition. Both DMPA and Levonorgestrel increase cervical mucus viscosity, making it more difficult for sperm to penetrate and reach the egg (Nakano *et al.*, 2015). This physiological change could contribute to lower fertilization rates and, ultimately, a reduced number of offspring per pregnancy.

The results of this study are consistent with findings from previous research on the reproductive effects of progestin-based contraceptives. Hu *et al.*, 2012 reported that female rodents treated with DMPA exhibited prolonged anovulation, leading to lower pregnancy rates and reduced litter sizes. Similarly, Endler *et al.* (2022) found that Levonorgestrel-treated rodents exhibited delayed implantation and increased rates of embryonic resorption, which could explain the reduction in litter size observed in the present study. The long-term effects of DMPA have also been shown to extend beyond the treatment period, often delaying the return to normal fertility post-treatment (Kaunitz, 1994; Damtie *et al.*, 2023).

These findings have several implications for reproductive biology and contraceptive research. The significant suppression of mating success and fecundity observed in this study suggests that both DMPA and Levonorgestrel may be effective in controlling reproduction, with DMPA exerting a more profound effect. This has potential applications in wildlife population control, contraceptive development, and fertility management. Future research should focus on assessing the reversibility of these effects by determining how long it takes for normal reproductive function to resume after treatment cessation. Additionally, further investigation into the molecular pathways affected by these contraceptives could provide deeper insights into their mechanisms of action. Studies comparing the effects of these contraceptives across different mammalian models would also be valuable in understanding species-specific variations in response to hormonal contraception.

### Study Limitations and Future Directions

While this study provides valuable insights, certain limitations should be noted. The use of a rodent model, though relevant for studying hormonal regulation, may not fully replicate human reproductive physiology. Additionally, the study did not evaluate long-term effects post-treatment. Future studies should include longer post-treatment cyclicity assessing recovery timelines for reproductive parameters, and the molecular mechanisms underpinning these effects.

## CONCLUSION

Overall, this study demonstrates that both DMPA and Levonorgestrel significantly altered the estrus cycle and fecundity. In addition, DMPA exerts a more profound and sustained inhibitory effect on several estrus cycles post-treatment. These findings highlight the need to further explore the reproductive health implications of frequent and prolonged usage of these contraceptives on women, especially those with a future plan of having their biological children. It also underscores the importance of counselling contraceptive users about potential delays in fertility recovery.
